# The roles of theaflavins in reducing dentin erosion

**DOI:** 10.1038/s41598-023-35382-3

**Published:** 2023-06-09

**Authors:** Jing Guo, Mingqi Yang, Mengna Hu

**Affiliations:** 1 Department of Dental General and Emergency, The Affiliated Stomatological Hospital of Nanchang University, No. 688 Honggu North Road, Honggutan District, Nanchang, 330038 People’s Republic of China; 2 The Key Laboratory of Oral Biomedicine, Nanchang, Jiangxi Province People’s Republic of China; 3 Jiangxi Province Clinical Research Center for Oral Diseases, Nanchang, People’s Republic of China; 4 State Key Laboratory of Food Science and Technology, Nanchang University, Nanchang, 330047 People’s Republic of China

**Keywords:** Dental caries, Cytokines, Enzymes

## Abstract

This study aimed to evaluate the effect of theaflavins [TFs] on the process of dentin erosion and investigation the potential mechanism. For erosion kinetics of the dentin, 7 experimental groups (n = 5) treated with 10% ethanol [EtOH] (negative control) are erosion for 1, 2, 3, 4, 5, 6, and 7 d erosion cycles (4 cycles/d). For the effect of TFs on dentin erosion, 6 experimental groups (n = 5) were treated with 1% epigallocatechin gallate [EGCG], 1% chlorhexidine [CHX], 1%, 2%, 4%, and 8% TFs for the 30 s and then subjected to erosion cycles (4 cycles/d for 7 d). The erosive dentin wear (μm) and surface morphology were evaluated and compared by laser scanning confocal microscope and scanning electron microscopy. The matrix metalloproteinase inhibition effects of TFs were investigated using in situ zymography and molecular docking. TFs-treated collagen was investigated by ultimate microtensile strength, Fourier-transform infrared spectroscopy, and molecular docking. Data were analyzed by ANOVA, Tukey’s test (*P* < 0.05). The TFs-treated groups (7.56 ± 0.39, 5.29 ± 0.61, 3.28 ± 0.33, and 2.62 ± 0.99 μm for 1%, 2%, 4%, and 8% TFs) had significantly lower erosive dentin wear than the negative control group (11.23 ± 0.82 μm), and the effect was concentration-dependent at low concentrations (*P* < 0.05). TFs inhibit matrix metalloproteinase [MMP]. Moreover, TFs crosslink dentin collagen and cause hydrophilic changes in dentin collagen. TFs preserve organic matrix within the demineralized dentin by inhibiting MMP activity and simultaneously improving collagen’s resistance to enzymes, both of which contribute to preventing or slowing down the progression of dentin erosion.

## Introduction

The effects of carbonated beverages on dental hard tissues have been extensively studied in the past decade, and a meta-analysis of studies evaluating dental erosion and diet showed that carbonated beverages were associated with an increased risk of dental erosion^1^. Due to the lack of obvious early symptoms and the poor recognition of patients with dental erosion, clinically, dental erosion is usually detected at a late stage^2,3^. In the late stage of dental erosion, dentin is exposed, leading to hypersensitivity, pulp destruction, and even tooth fracture.

For enamel with simple structure and composition, well-established treatment options have been proposed, such as the use of fluoride which can effectively treat enamel erosion and promote its remineralization^4,5^. Dentin is structurally complex, poorly mineralized, and contains more collagen. In the case of dentin erosion, the inorganic components from the peritubular/intertubular junction first dissolve, followed by the peritubular dentin is lost, which causes the dentin tubules to widen and ultimately demineralized organic matrix is detectable. Previous studies have shown that the dentin organic matrix can prevent ion diffusion and reduce the damage of acid to dentin^6,7^. However, dentin organic matrix is susceptible to degradation by MMP mainly including MMP-2, MMP-8, and MMP-9^8^. This degradation occurs because MMP are inactive in saliva and dentin, but in an acidic environment, MMP are activated and initiate their proteolytic action, thus contributing to the progression of erosive wear^9^.

Strategies, including application of collagen cross-linking and MMP inhibitors. Treatments may include the application of chlorhexidine (CHX), which has been found to be particularly beneficial of dentin erosion, but have cytotoxic effect^10,11^. There is a growing body of evidence suggesting that commercial green tea and green tea extract (epigallocatechin gallate, EGCG) rinses can reduce erosive and erosive/abrasive dentin wear caused by extrinsic acids^12,13^. However, few studies have examined the effects of black tea derivatives/active compounds on dentin erosion.

**Figure 1:**
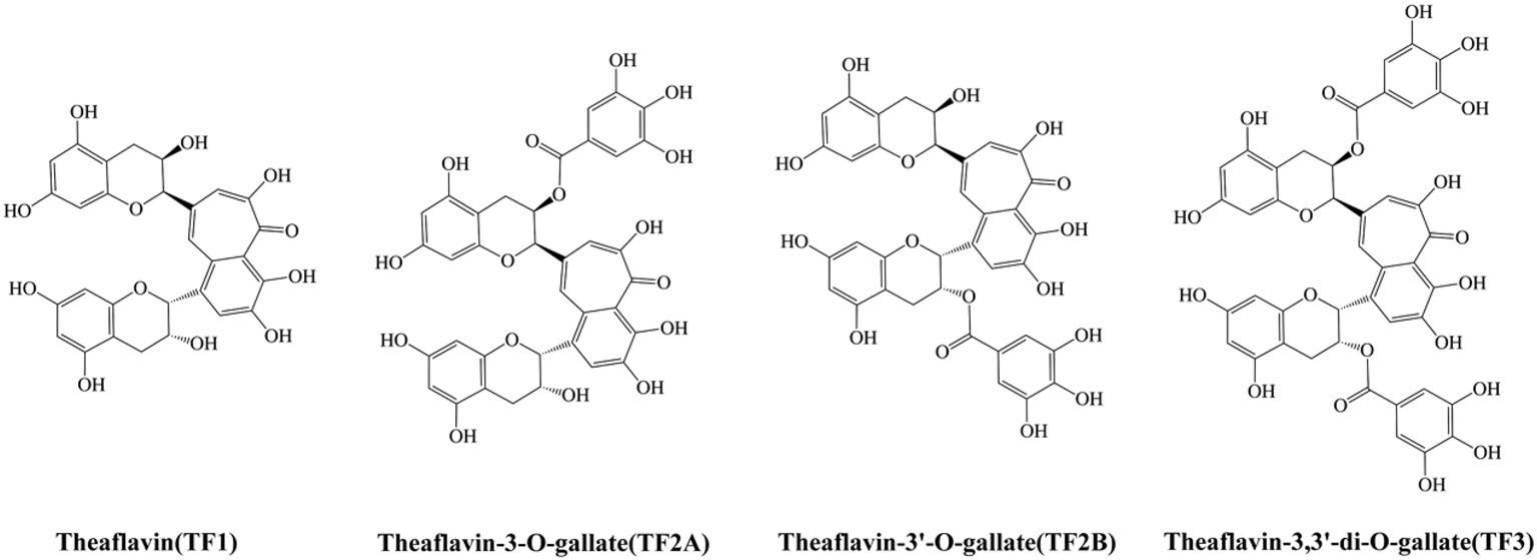
Schematic diagrams of molecular structures of theaflavins (TFs).

TFs are polyphenolic compounds found in black tea and exhibit a variety of health benefits, including antimicrobial^14^, anti-HIV^15^, anticancer^16^, cholesterol-lowering^17^, and low toxicity to normal cells^18^. In particular, from the chemical structure aspect, TFs have aromatic rings similar to EGCG attached with abundant phenol hydroxyl groups as well as galloyl groups and mainly contain four structures of dimers (Fig. 1). Theoretically, TFs should reduce dentin erosion in a similar way as EGCG. In addition, previous reports have shown that TFs have poor systematic bioavailability. It is reported that only a small amount of TFs can be detected in the plasma and urine samples of healthy volunteers after 2 h of consumption of 700 mg mixed TFs^19^. In this text, TFs were directly applied to the oral care process to provide a new idea for its practical application.

Therefore, this study aimed to evaluate the effects of TFs on the process of dentin erosion and investigation the potential mechanism, including its ability to inhibit MMP and cross-link collagen. The null hypothesis was that TFs would not be able to (i) reduce dentin erosion, (ii) inhibit MMP and (iii) cross-link dentin collagen via chemical interactions.

## Materials and methods

### Specimen preparation

Non-carious human molars were collected with no associated patient identifiers, collection protocol determined as not human subject research, the study was approved by the Independent Ethics Committee of the Affiliated Stomatological Hospital of Nanchang University and all methods were carried out in accordance with relevant guidelines and regulations. All collected molars were informed consent by subjects and/or their legal guardian(s). All methods were carried out in accordance with relevant guidelines and regulations. The extracted human third molars were used within 1 month and preserved in 0.01 M phosphate-buffered saline (pH7.3, ZSGB-BIO Ltd., CHN) at 4 °C. Seventy-nine dentin blocks (6 mm × 2 mm × 2 mm) were obtained from the cervical with a precision cutting machine (Iso Met™ 1000, Buehler Ltd., USA) under water cooling. The test surfaces of the blocks were individually burnished with a series of silicon carbide papers (400, 600, and 1200 grit; 3 M Ltd., USA) for 1 min and cleaned with an ultrasonic cleaning machine for 10 min. Finally, sixty-five dentin blocks with surface microhardness between 60 and 70 (Vickers diamond, 100 g, 10 s; VH1202, Buehler Ltd., USA) were used. Half of the test surface (distal pulp surface) was covered using anti-acid nail varnish. All dentin blocks had to be kept moist to avoid the contraction of the dentin organic matrix during the experiment.

The blocks were first exposed to artificial saliva for 1 h to allow the acquired salivary pellicle to form on the dentin surfaces^20^, the artificial saliva was mixed according to the formulation described by Klimek J et al^21^.

### The erosion kinetics of the dentin

Thirty-five dentin blocks were treated with 10%EtOH (negative control and solvent for EGCG, CHX, and TFs) for the 30 s and then immersed in artificial saliva for 2 h. The blocks were randomly assigned to seven groups based on different erosion times (n = 5):1, 2, 3, 4, 5, 6, and 7 d erosion cycles. In one erosion cycle, the blocks were exposed to 5 ml of phosphoric acid aqueous solution (pH = 2.3) for 5 min at 37 °C, cleaned with an ultrasonic cleaning machine for 10 s, and immersed in 5 ml of artificial saliva for 1 h. After the 4 erosion cycles per day were completed, the dentin blocks were soaked in artificial saliva overnight at 37°C^22^. Finally, the acid-resistant nail polish was carefully removed with a forcep before testing.

**Figure 2:**
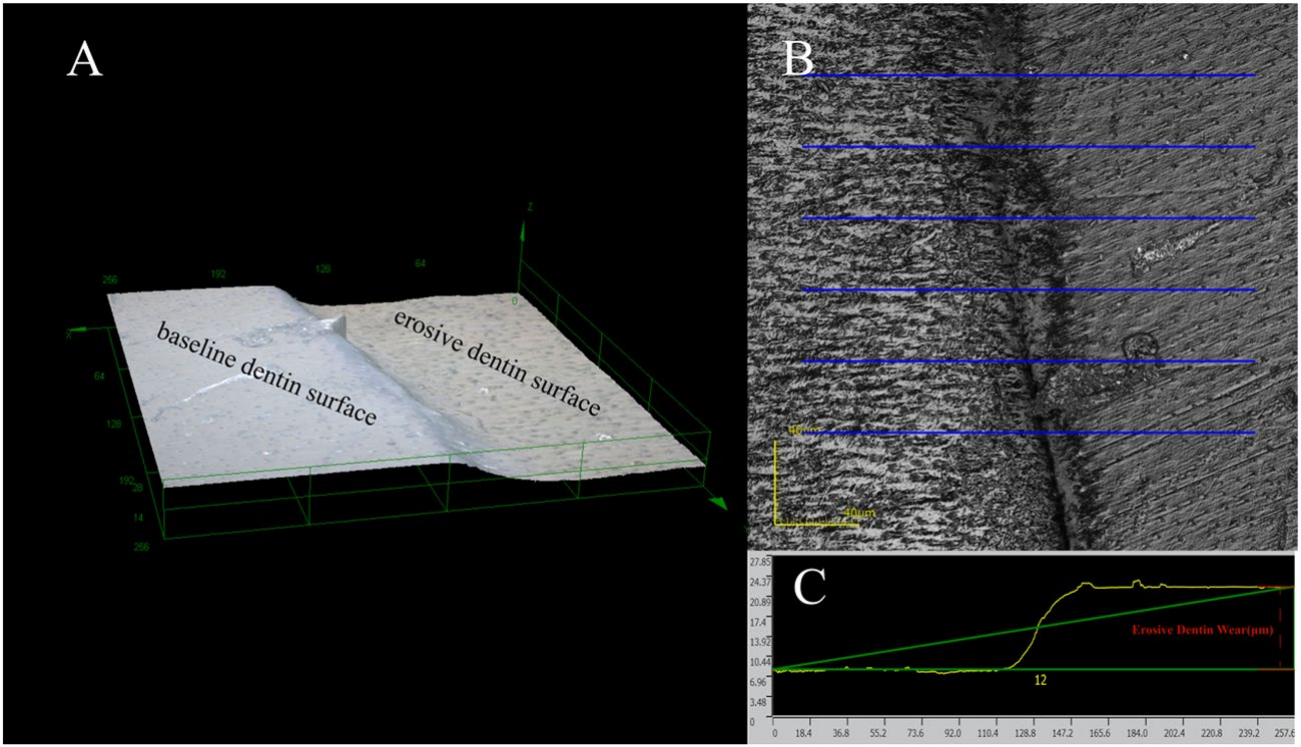
The diagram of erosive dentin wear evaluation. (**A**) 3D image of dentin surface by LEXT OLS 4000; (**B**) place trace lines on dentin surface; (**C**) determine erosive dentin wear (μm) using the two-point method in LEXT OLS 4000 software.

The erosive dentin wear(μm) evaluation was according to the method described by Paepegaey AM et al^23^. The specimens were analyzed on a laser scanning confocal microscope (LEXT OLS4000, Olympus Ltd., UK). The instrument can scan the sample surface with a laser, capture 3D data sets to generate 3D images (Fig. 2A), and allow 3D measurement. A single image of each block’s surface was captured using the × 50 objective. After data gathering, the data were processed following these steps with LEXT OLS4000 software.

i. six “traces” were placed on the sample surface (Fig. 2B).
ii. The height difference between the baseline dentin surface and the eroded dentin surface was measured using the two-point method in the software (Fig. 2C), and the average value was determined as eroded dentin wear (μm).

### Effects of TFs on dentin erosion

Thirty dentin blocks were randomly assigned to 6 groups based on the treat solutions (n = 5):1% EGCG(shanghai yuanye Ltd., CHN)), 1% CHX(Macklin Ltd., CHN), 1%, 2%, 4%, and 8% TFs(Bomei Ltd.,CHN). EGCG and CHX were used as positive control. During the experiment, these blocks experienced 7 consecutive days of erosion cycle. After brief drying, the blocks were treated with a corresponding solution for 30 s using a microbrush. The blocks were then immersed in artificial saliva for 2 h, followed by a daily erosion cycle. The erosion cycle scheme and the calculated method of erosive dentin wear (μm) as mentioned in Sect. 2.2.

After completing erosive dentin wear (μm), randomly select one block from each evaluation group for scanning electron microscopic (SEM) observation. The blocks were fixed with 2.5% glutaraldehyde buffer containing 0.1 M sodium cacodylate for 2 h and dehydrated with ethanol (33%, 67%, 85%, 95%, and 100% for 30 min). Subsequently, the blocks were subjected to air drying overnight. The specimens were gold sputtered and observed using SEM (Phenom LE, Phenom Scientific, NLD) at 15 kV.

### Interaction between TFs and MMP

#### In situ* zymography*

Additional fourteen dentin blocks were assigned into 7 groups based on the different treatment solutions (two blocks/group): untreated(10% EtOH), 1% EGCG, 1%CHX, and 1%, 2%, 4%, or 8% TFs were used for in situ zymography assessment. The fluorescein-conjugated gelatin mixture (E-12055, Invitrogen, USA) was prepared according to the manufacturer’s protocol before use. The dentin blocks were ground with 600 and 1200-grit silicon carbide papers under flowing water to approximately 50 μm thickness and treated with the corresponding protocols for 30 s. Moreover, the blocks were submerged in 5 ml of a phosphoric acid aqueous solution for 5 min, rinsed with deionized water for 1 min, and spread onto glass slides. The fluorescein-conjugated gelatin mixture (3 μl) was then dropped on each block and incubated for 24 h in a humidified chamber at 37 °C away from light. Each glass slide containing the blocks was covered with coverslips. Furthermore, every block was tested with a confocal laser scanning microscope (CLSM) (Zeiss LSM800, Carl Zeiss AG Ltd., Germany) in fluorescence mode (40 × objective lenses of 0.75 NA) at excitation/emissions of 488/530 nm.

Three images obtained from the same z-layer of each dentin block were randomly captured. All images (n = 6) were analyzed and quantified using NIH Image J 1.8.0 software (Bethesda, MD, USA) to examine the hydrolysis of the fluorescein-conjugated gelatin substrates. According to the value of the relative green fluorescence, this process can imply the activity of dentin endogenous MMP (mainly MMP-2 and MMP-9)^24^.

#### Molecular docking between TFs and MMP

The interactions between MMP-2(PDB IDs:1QIB)/MMP-9 (PDB IDs: 2OW0)^25,26^ and TFs were simulated using AutoDock 4.2 (https://autodock.scripps.edu/)software^27^. The crystal structures of MMP-2 and MMP-9 were selected from the Protein Data Bank (PDB) database (https://www1.rcsb.org/). The macromolecule was set as a rigid static entity limited in a three-dimensional mesh box for completely covering the active site of the macromolecule. The ligand in the box was treated as a flexible molecule with translational, orientational, and torsional degrees of freedom. The most probable docking location and the lowest binding energy were identified using the Lamarckian genetic algorithm. The initial population for the Lamarckian genetic algorithm was set as 150 individuals, the maximal number of energy evaluations was 2.5 × 10^6^, the maximal number of energy generation was 27,000, the selected number for docking rounds was 100, and the free binding energy values of the ligands were calculated using a semiempirical free energy force field^28^. The macromolecule–ligand interaction analysis used PLIP(Protein–Ligand Interaction Profiler)^29^. Visualization of the macromolecule–ligand complex was performed using the Open-Source PyMOL Molecular Visualization System (Version 2.5, Schrödinger LLC., USA) (https://autodock.scripps.edu/).

### Interaction between TF and collagen

#### Ultimate microtensile strength of collagen

Dentin strips (1 mm × 1 mm × 10 mm) were cut from human third molars' crowns and then randomly divided into 5 groups based on the different treatment solutions (n = 10): 10% EtOH, 1%, 2%, 4%, and 8% TFs. The dentin strips were demineralized with 10% H_3_PO_4_ for 72 h, then rinsed with deionized water for 7 d to gain dentin collagen^30^. After rinsing, the strips were immersed in treatment solutions for 30 s at room temperature. Subsequently, the strips were thoroughly rinsed with deionized water to remove the residual treatment solution.

To determine the ultimate microtensile strength (μUTS), each strip was glued with cyanoacrylate adhesive (ergo. 5180, Kisling Ltd, Germany) to stick each strip to the two free sliding parts of the fixture, which was mounted on a microtensile tester (MTT, Bisco, USA) and subjected to microtensile testing at a crosshead speed of 1 mm/min until rupture occurred^31^. The μUTS (MPa) was calculated according to the following formula^30^:

$$\mathrm{\mu UTS}=\frac{F}{S}$$

where F is the maximum load (N), and S is the sample Cross-sectional area (mm^2^).

#### Fourier transform infrared spectroscopy (FT-IR)

Use a water-cooled low-speed diamond saw to remove enamel from the teeth. Slice the obtained dentin block along the mesial-distal direction and wet grind it into a block with a thickness of 50 μm, which resulted in a total of two dentin blocks from one molar. The dentin blocks were demineralized with 10% H_3_PO_4_ for 60 min, then rinsed with deionized water for 7 d to gain dentin collagen blocks. TFs-treated dentin collagen block was prepared at room temperature by immersed of collagen into 1%TFs for the 30 s. Untreated collagen block served as the control. The treated and untreated collagen block dried for 48 h in a desiccator under a vacuum. The dried dentin collagen blocks were scanned using a Fourier transform infrared spectroscopy (FT-IR) spectrometer (Nicolet IS10, Thermo Fisher Scientific Inc., USA) in transmission mode using the ATR technique in the wavenumber range 4000–500 cm^−1^ with a resolution of 4 cm^−1^ and 64 scans.

#### Molecular docking between TFs and collagen

In this study, type-I collagen(PDB IDs: 1QSU)^32^, which is the major organic component of demineralized dentin, served as the receptor for molecular docking with TFs, Docking simulations were performed using the AutoDock program package according to the method mentioned above.

The interactions between type-1 collagen and the ligand structures were studied using AutoDock docking software, and the most probable docking location and the lowest binding energy predicted were screened. Docking parameters, calculation of the free binding energy values of the ligands, macromolecule–ligand interaction analysis, and visualization of collagen–ligand complex as mentioned in Sect. 2.4.2, and the selected number for docking rounds was 50.

### Statistical analysis

Kolmogorov–Smirnov and Levene’s tests assessed the normal distribution of error equal variance assumption. Statistical analysis of the erosive dentin wear, in situ zymography, and ultimate microtensile strength data were performed using one-way analysis of variance (ANOVA) and Tukey’s test. The data were analyzed using the Statistical Package for the Social Sciences (SPSS) statistical software package (SPSS 25.0 for MAC, SPSS, Chicago, IL, USA), and all statistical analyses were performed at a significance level of 0.05.

## Result

### The erosion kinetics of the dentin

**Figure 3:**
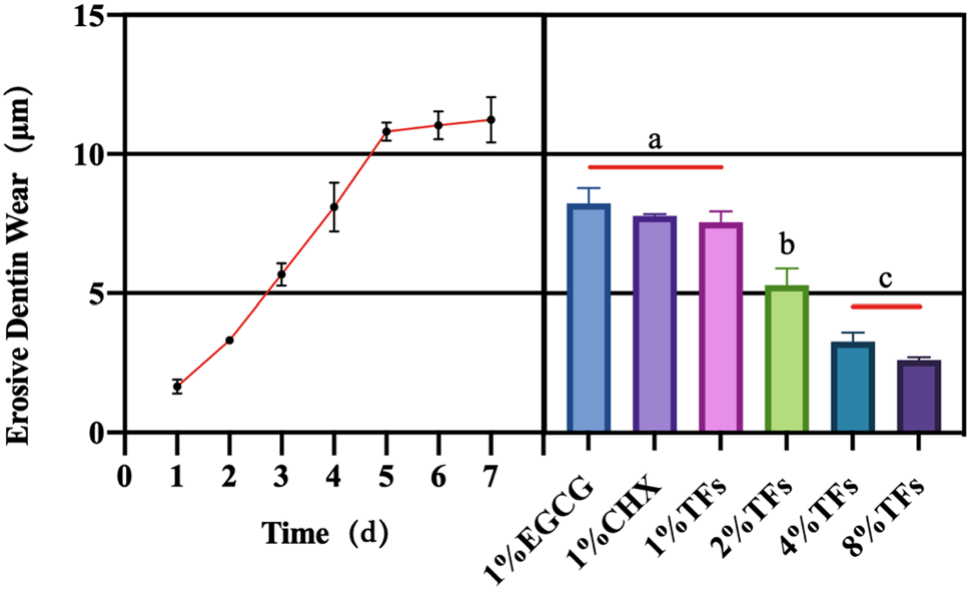
(Left) is the erosion curves of dentin for 7 d. (Right) is the erosive dentin wear (μm) for different treated groups. Means with different letters are significantly different (*P* < 0.05).

The erosive dentin wear(μm) of blocks at the different times respectively is 1.65 (0.25), 3.32 (0.06), 5.67 (0.41), 8.09 (0.87), 10.80(0.33), 11.04 (0.50) and 11.23 (0.82) after erosion cycles. Figure 3(left) shows the erosion curves of dentin. From the fifth day, the rate of dentin demineralization slowed significantly and reached a demineralization plateau.

### Effects of TFs on the dentin erosion

Figure 3(right) shows the erosive dentin wear (μm) of the blocks treated with different solutions after the samples were subjected to erosion cycles. The erosive dentin wear(μm) of blocks treated with different solutions respectively is 8.23 (0.55), 7.77 (0.07), 7.56 (0.39), 5.29 (0.61), 3.28(0.33) and 2.62 (0.99) after erosion cycles. The erosive dentin wear(μm) was reduced in EGCG/CHX/TFs groups compared with the negative control (10% EtOH) (all *P* < 0.05). 1% EGCG/CHX/TFs groups showed similar erosive dentin wear (P > 0.05). At low concentrations (< 2%), the reducing erosive dentin wear (μm) effect of TFs was concentration-dependent (*P* < 0.05). The specimens treated with 4% and 8% TFs exhibited the least erosive dentin wear (all *P* < 0.05).

**Figure 4:**
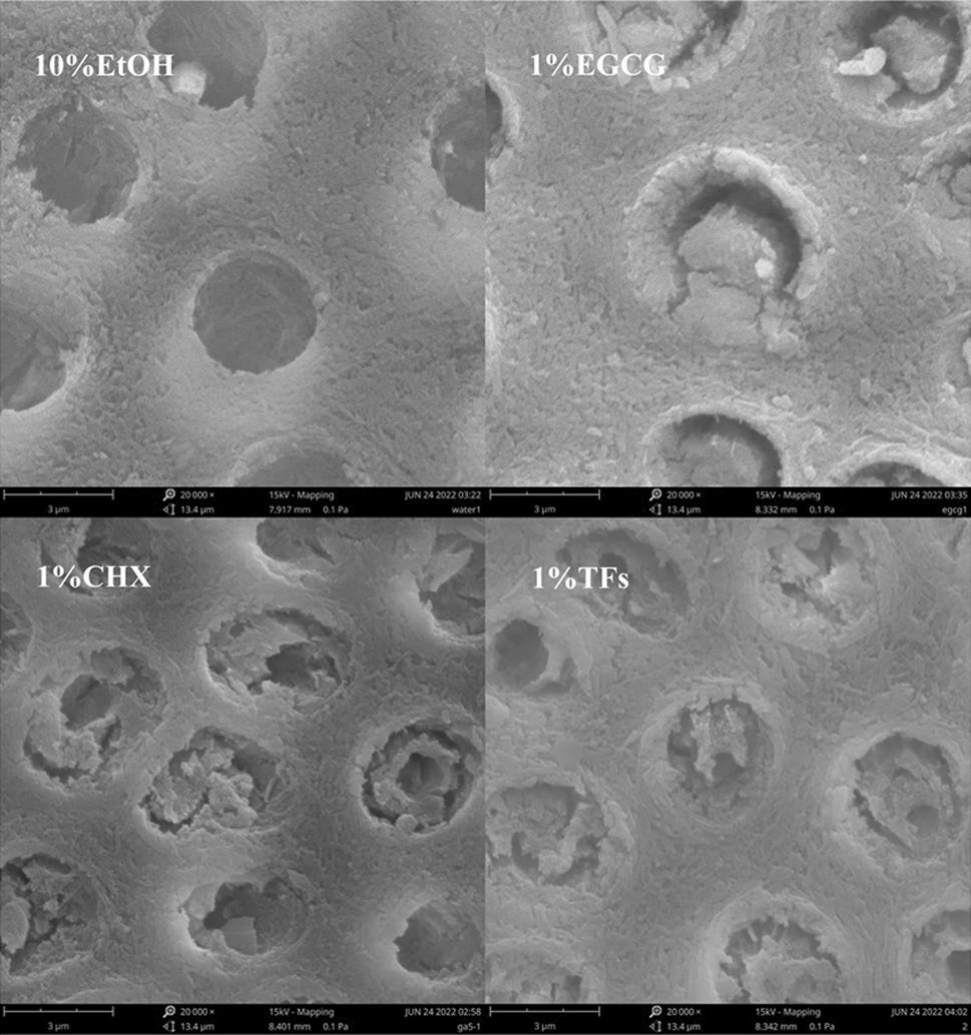
Representative SEM images of the dentin surface after the erosion cycle (× 20,000).

The SEM images (Fig. 4) showed that the occlusion of dentinal tubules was obvious in the 1% EGCG/CHX/TFs treated group, and the collagen structure was preserved, whereas most of the dentinal tubules in the negative control (10%EtOH) group were completely exposed, and the collagen structure was completely destroyed. Although a similar erosion state was observed on the surface of dentin blocks in each group, the negative control group showed more severe erosion.

### Interaction between TFs and MMP

**Figure 5:**
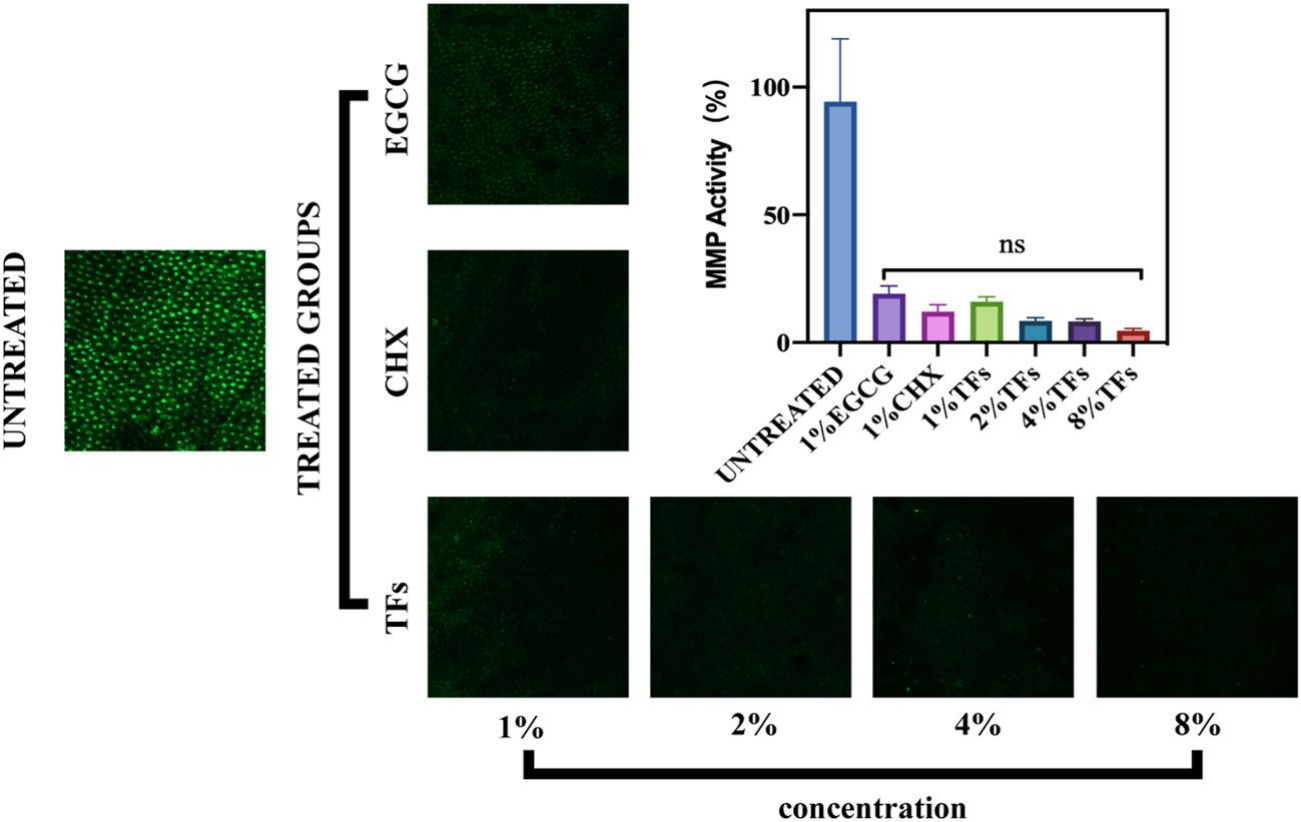
Representative images (left) of confocal laser scanning microscopy (CLSM) and relative green fluorescence intensity representing endogenous MMP activity or inhibition (right graph) of the controls and treated dentin blocks after exposure to the phosphoric acid aqueous solution for 5 min, and 24 h of incubation in the quenched fluorescein-labeled gelatin. ns: not significant(p > 0.05).

The endogenous MMP activity in the untreated and treated groups was assessed by CLSM and in situ zymography. The representative images (left) by in situ zymography and relative green fluorescence intensity of the endogenous MMP activity (right) in the untreated and treated groups showed in Fig. 5. The untreated group showed the largest green fluorescence intensity, which indicates that MMP has the strongest activity. The green fluorescence intensity of the EGCG, CHX, and TFs groups was significantly lower than that of the untreated control group (all *P* < 0.05). Moreover, the activity of MMP could not be significantly decreased with the increase in TFs-treatment concentration (P > 0.05).

**Figure 6:**
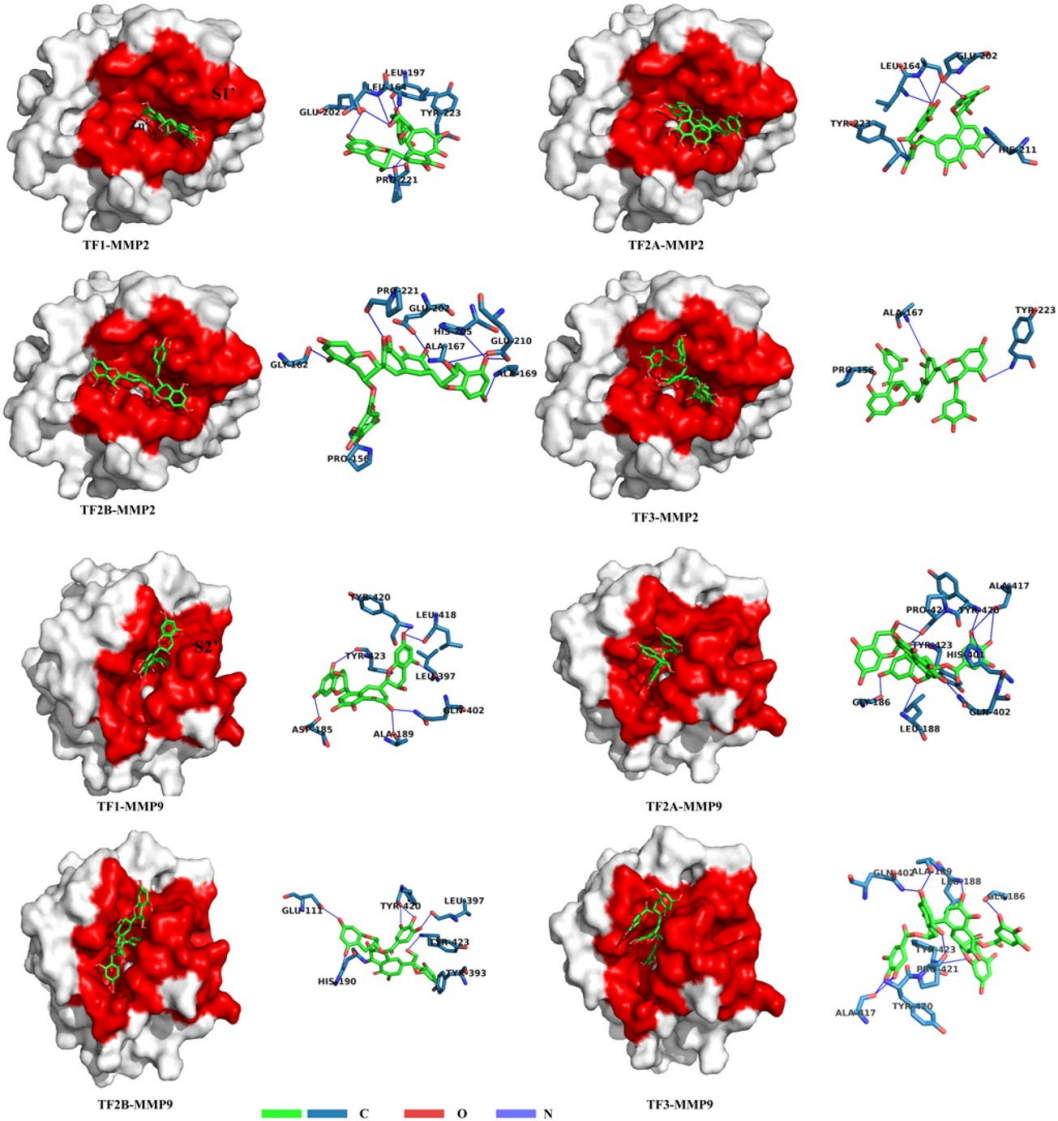
Molecular docking. Representative illustrations of the docked MMP-2 (1QIB)-TFs(TF1, TF2A, TF2B, TF3) complex and the MMP-9 (2OW0)-TFs(TF1, TF2A, TF2B, TF3) complex. Left: Contour visualizations. The MMP-2 and MMP-9 structure are shown as white surfaces, and the specificity pockets S1’ and S2’ are marked in red. Right: Binding modes for TFs(TF1, TF2A, TF2B, TF3) with the active site residues. Hydrogen bonds are shown in blue dashed lines.

Docking results provided us with information about its mechanism, including binding mode, key amino acids, and intermolecular forces, the docking data are summarized in Table 1 and Fig. 6.The minimum binding energy from 100 docking results between MMP-2 and TF1, TF2A, TF2B, and TF3 respectively is -7.77, -5.17, -5.93, and -3.92 kcal/mol. Moreover, the minimum binding energy between MMP-9 and TF1, TF2A, TF2B, and TF3 respectively is -10.48, -9.66, -10.25, and -10.31 kcal/mol. The binding energies of all complexes are thermodynamically favorable, indicating that these interactions can proceed spontaneously.

**Table 1:** Molecular docking result of TFs with MMP.

Ligand-macromolecular	Hydrophobic interaction	Hydrogen bonds	Salt bridges	π-π-Stacked
TF1-MMP2	LEU164, HIS166, VAL198, HIS201, ILE222, TYR223	LEU164, ALA165, LEU197, GLU202, ALA220, PRO221, TYR223	HIS211	HIS201
TF2A-MMP2	HIS166, PRO221, ILE222, TYR223	LEU164, ALA165, GLU202, HIS211, TYR223		HIS211
TF2B-MMP2	LEU163, ALA169, ILE222	PRO156, GLY162, ALA167, ALA169, GLU202, HIS205, GLU210, PRO221,		
TF3-MMP2	TRY155, LEU164, HIS166, TRY223	PRO156, ALA167, TYR222	HIS211	
TF1-MMP9	LEU188, VAL398, HIS401, LEU418, TYR423	APS185, ALA189, LEU397, GLN402, LEU418, TYR420, TYR423		TYR393, HIS401
TF2A-MMP9	LEU187, LEU188, HIS401, TYR423	GLY186, LEU188, HIS401, GLN402, ALA417, TYR420, PRO421, TYR423	HIS401	HIS411
TF2B-MMP9	PHE110, TYR393, VAL398	GLU111, HIS190, TYR393, LEU397, TYR420, TYR423		HIS401
TF3-MMP9	LEU188, TYR393, TYR423	GLY186, LEU188, ALA189, 402GLN, ALA417, TYR420, PRO421, TYR423	HIS401

### Interaction between TFs and dentin collagen

The strips treated with different concentrations of TFs exhibited significantly higher μUTS values (15.86 (3.19), 20.08 (5.29), 26.67(6.31), and 25.51(4.97) MPa for 1%, 2%, 4%, and 8%, respectively) than the control group(9.74 (2.03) MPa for 10% EtOH) (all *P* < 0.05). The μUTS value of 2%, 4%, and 8% TFs was significantly higher than those of 1% (all *P* < 0.05).

**Figure 7:**
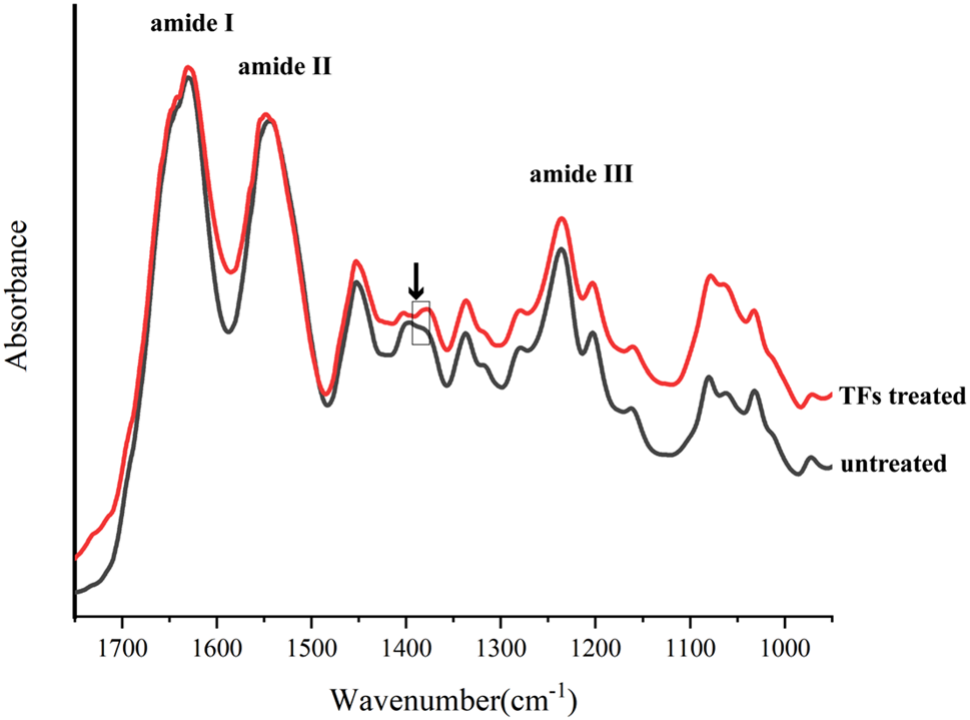
The FT-IR spectra of dentin collagen before and after TFs-treated.

Figure 7 shows the FT-IR spectra of dentin collagen before and after TFs-treated. The three peaks located at 1640, 1539, and 1235 cm^-1^ represent the characteristic amides I, II, and III bands of collagen, respectively. The decreased intensity of the band is observed at ~ 1400 ­cm^−1^ for TFs-treated collagen, and the broadening of amide I (~ 1640 cm^-1^) to lower wavenumbers was observed.

**Figure 8:**
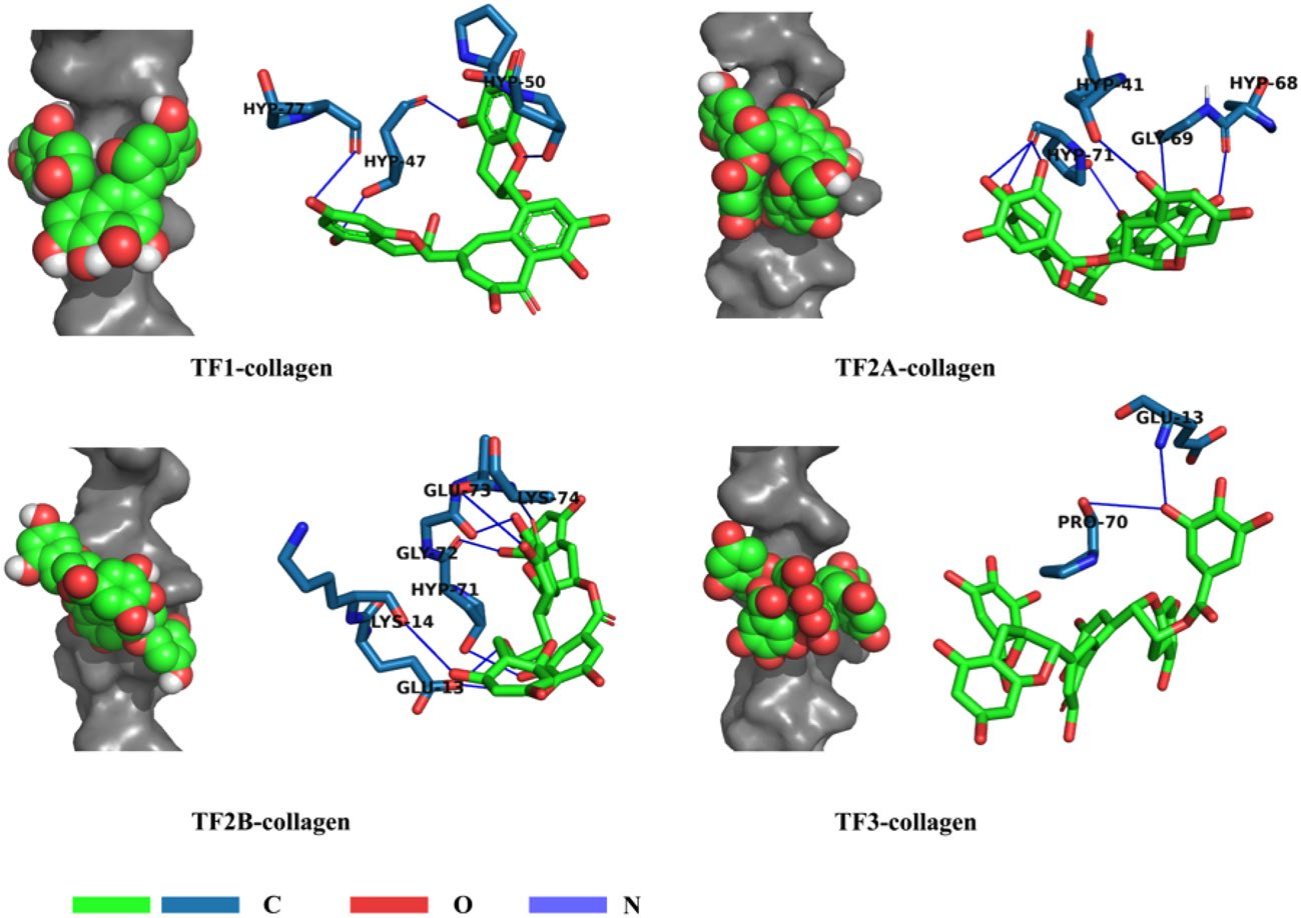
Representative illustrations of the docked dentin collagen (1QSU)-TFs(TF1, TF2A, TF2B, TF3) complexes. Left: Steric complementarity. Right: Visualizations of hydrogen bonding.

Docking results for collagen and TFs are summarized in Table 2 and Fig. 8, The minimum binding energy from 50 docking results between collagen and TF1, TF2A, TF2B, and TF3 respectively is -4.47, -4.43, -3.79, and -8.15 kcal/ mol, which suggests that these interactions are spontaneous. Then, we visualized the collagen-ligand complex (Fig. 8). All four dimers of TFs insert into a hydrophobic cavity on the surface of collagen, as a result of its structural flexibility and alignment with complementary functional sites on collagen (left). It is known from the results that TFs formed hydrophobic interactions and hydrogen bonds with key amino acid residues on the collagen surface, the 3D visualization of docking sites (right) displays the hydrogen bonds formed between key amino acid residues of collagen and four dimers of TFs.

**Table 2:** Molecular docking result of TFs with collagen receptor.

Ligand-macromolecular	Hydrogen bonds	Hydrophobic interactions
TF1- collagen	HYP47, HYP50, HYP77	HYP47, PRO49, PRO76, PRO79
TF2A- collagen	HYP41, HYP68, GLY69, HYP71	HYP38, PRO70, HYP71
TF2B- collagen	GLU13, LYS14, HYP71, GLY72, GLU73, LYS74	PRO70, HYP71
TF3- collagen	GLU13, PRO70	HYP38, HYP68, PRO70

## Discussion

Oral clinicians and researchers have paid great attention to dental erosion and caries caused by the excessive consumption of carbonated beverages. They are exploring the optimal strategy to reduce dentin erosion and caries using minimally invasive treatments^33^. To our knowledge, this work is the first to show that TFs can inhibit dentin erosion.

The erosion power of beverages on natural teeth is thought to be related to several factors, such as type of acid content, pH, titratable acidity, ion concentration, and calcium chelating properties^34^. Although all carbonated beverages contain a small amount of carbon dioxide in a solution^35^, studies have shown that sparkling water is less corrosive because no other acids are added to its content, and demineralization on teeth is less when it comes in contact with sparkling water compared with other carbonated beverages^36^. The structural units of the tooth hard tissue can still be identified even after 7 days of carbonated water exposure^37^. The above research shows that the erosion potential of carbonated beverages may be mainly due to the low pH value caused by the addition of additional acids, such as phosphoric acid in Cola. Therefore, phosphoric acid aqueous solution (pH = 2.3) was used as an erosion solution to simulate the most popular carbonated beverage: Cola. Moreover, CHX and EGCG were selected as positive controls, which have been shown to inhibit dentin erosion and are commonly used in oral studies. The lowest concentrations known to inhibit MMP-2 and MMP-9 by chlorhexidine were 1 μg/mL and 20 μg/mL^13^, but the lowest concentrations described above were derived under the condition that chlorhexidine directly interacts with MMP for 30 min, increasing its concentration is necessary to achieve its inhibition of MMP within a clinically relevant time. The concentration of CHX was derived from an already commercially available varnish (Cervitec) that has been shown to reduce dentin material loss over a clinically relevant time^38^, and the same concentration was adopted to EGCG and TFs to avoid unnecessary errors. Meanwhile, the effects of higher concentration TFs were tested to not risk an effect below the desired.

In this study, we attempted to unmask the process of dentin erosion by carbonated beverages so first conducted erosion kinetic studies. The results showed that although only a small amount of phosphoric acid was added to the cola, it caused severe dentin erosion. In addition, as seen in the previous studies^39^, dentin demineralization rates slowed after prolonged erosion, and a demineralization plateau was reached ( the depth did not change more than 30% from one-time point to another until the last measurement). This phenomenon might be associated with the protective role of the organic matrix in dentin demineralization^40^, which would retard the intertubular dentin demineralization and retains its height and volume. Thus, the present study demonstrated again that maintaining a demineralized organic matrix is essential to prevent or slow down dentin erosion.

Subsequent results support those of previous studies suggesting the potential of MMP inhibitors in preventing or slowing down the progression of dentin erosion^41,42^. Any treatment protocol using MMP inhibitors can result in dentine erosion less than the demineralized platform (Fig. 3), suggesting a protective effect of MMP inhibitors on the demineralized dentin matrix. And, TFs exhibited a similar performance as the well-known MMP inhibitors (EGCG and CHX). This phenomenon could be due to TFs possessing the ability to inhibit MMP activity. To illustrate MMP inhibition by TFs, in situ zymography and molecular docking were used. In situ zymography shows the treated dentin with TFs significantly decreased the green fluorescence intensity (Fig. 5), which indicated that the activity of MMP (mainly MMP-2 and MMP-9) could be effectively reduced by TFs. Their interaction was determined by two common characteristics, i.e., van der Waals contacts in the active site cleft and hydrogen-bond interactions with S1′ and S2′ active site. The main sub-sites of substrate recognition in MMP-2 and MMP-9 are specific pockets S1′ and S2′, which are considered to be the most important in substrate and inhibitor specificity^43–45^. Computer simulations revealed that approximately eight residues (LEU164、HIS166、HIS201, GLU202, HIS211, PRO221, ILE222, TYR223) in MMP-2 and seven residues (APS188, TYR393, HIS401, TYR402, HIS411, TYR420, TYR423) in MMP-9 surround the TFs and forms van der Waals forces, hydrophobic interactions, hydrogen bonds, salt bridges, and π—π stacking, contributing binding energy to the formation of the complex. There are related reports that amino acid residues HIS201, HIS 205, and HIS 211 combine with Zn^2+^ to form the active site of MMP-2^46^, amino acid residues HIS 401, HIS 405, and HIS411 combine with Zn^2+^ to form the active site of MMP-9^45^, and from Fig. 8, it can be observed that all four dimers of TFs can insert into the zinc-containing active pocket of MMP-2 and MMP-9, from which it can be deduced that TFs can enter the active sites of MMP-2 and MMP-9 and bind to HIS201, HIS211, and HIS401, respectively HIS411 interacted with each other to form van der Waals, hydrophobic interactions, hydrogen bonds, salt bridges, and π-π stacking, indicating their potential mechanism of enzyme inhibition for MMP-2 and MMP-9^45,47–49^, and the mechanism is competitive inhibition. Therefore, it can be concluded that TFs bind specifically to the MMP-2 and MMP-9 catalytic domains, disrupting their recognition and binding to collagen, and leading to the inhibition of enzyme activity. All the results indicate TFs can reduce dentine erosion by inhibiting MMP activity. Therefore, the first and second null hypothesis was rejected.

Interestingly, the in situ zymography showed that increasing the concentration of TFs did not significantly reduce MMP activity (*P* < 0.05), but the effect of higher concentrations of TFs (2%, 4%, 8%) on dentin erosion was significantly better than 1% EGCG, CHX, and TFs. Moreover, cross-linking collagen can improve the mechanical properties of dentin and increase its resistance to enzymatic degradation^50^. Thus, the interaction between TFs and collagen was further investigated.

Previous studies have shown that the degree of collagen crosslinking is positively correlated with its tensile strength^30,51^. Therefore, tensile strength was used to investigate the effect of TFs application on the mechanical properties of the dentin organic matrix. According to the present results, TFs could significantly increase μUTS of the dentin organic matrix. This suggests that TFs have the potential to crosslink collagen. For further demonstration, TFs-treated collagen was examined by FT-IR spectrum. In the FT-IR spectrum, the decreased intensity of the band at ~ 1400 ­cm^−1^, is caused by the dehydration of collagen fibers, indicating that a strong hydrogen bond is generated between collagen and TFs ^52–54^. The broadening of amide I also indicate hydrogen bonding between the amide group(-CONH_2_) and amino(-NH_2_) of dentin collagen and phenolic hydroxyl (OH) groups of TFs ^55,56^. The formation of hydrogen bonds is the main feature of cross-linking, and the above results indicate that TFs have the ability to crosslink dentin collagen, which contributes to the increased resistance of collagen fibers to enzymatic hydrolysis^57,58^. Meanwhile, the maintenance of amide I, II, and III peaks in FT-IR spectra illustrated that TFs did not disrupt the integrity of the collagen triple helix structure of dentin. The hydrogen bonds between TFs and collagen were further confirmed by molecular docking.

As shown in molecular docking simulations (Table 2 and Fig. 8), and four dimers of TFs insert hydrophobic cavities along the collagen surface, covering the recognition sites of collagen surface enzymes to provide direct physical protection to the collagen^26^. In addition, the hydrophobic chains of TFs interact with the hydrophobic regions of collagen, This hydrophobic interaction may also help to exclude water molecules from the collagen surface in the aqueous medium, which in turn helps to protect dentin collagen from MMP^59^. Accordingly, we proposed that TFs may via causing collagen crosslink, changes in hydrophilicity, and covering the enzyme binding sites on the collagen surface to the decreased enzymatic hydrolysis of collagen. Therefore, the third null hypothesis was rejected.

## Conclusion

In summary, the results of this study show that TFs have a superior effect in preventing or slowing down the progression of dentin erosion. And, we propose that TFs are a competitive inhibitor of MMP-2 and MMP-9 and may via causing collagen crosslink, changes in hydrophilicity, and covering the enzyme binding sites on the collagen surface to the decreased enzymatic hydrolysis of collagen.
